# RNA threading with secondary structure and sequence profile

**DOI:** 10.1093/bioinformatics/btae080

**Published:** 2024-02-10

**Authors:** Zongyang Du, Zhenling Peng, Jianyi Yang

**Affiliations:** Chongqing Key Laboratory of Big Data for Bio Intelligence, Chongqing University of Posts and Telecommunications, Chongqing 400065, China; MOE Frontiers Science Center for Nonlinear Expectations, Research Center for Mathematics and Interdisciplinary Sciences, Shandong University, Qingdao 266237, China; MOE Frontiers Science Center for Nonlinear Expectations, Research Center for Mathematics and Interdisciplinary Sciences, Shandong University, Qingdao 266237, China

## Abstract

**Motivation:**

RNA threading aims to identify remote homologies for template-based modeling of RNA 3D structure. Existing RNA alignment methods primarily rely on secondary structure alignment. They are often time- and memory-consuming, limiting large-scale applications. In addition, the accuracy is far from satisfactory.

**Results:**

Using RNA secondary structure and sequence profile, we developed a novel RNA threading algorithm, named RNAthreader. To enhance the alignment process and minimize memory usage, a novel approach has been introduced to simplify RNA secondary structures into compact diagrams. RNAthreader employs a two-step methodology. Initially, integer programming and dynamic programming are combined to create an initial alignment for the simplified diagram. Subsequently, the final alignment is obtained using dynamic programming, taking into account the initial alignment derived from the previous step. The benchmark test on 80 RNAs illustrates that RNAthreader generates more accurate alignments than other methods, especially for RNAs with pseudoknots. Another benchmark, involving 30 RNAs from the RNA-Puzzles experiments, exhibits that the models constructed using RNAthreader templates have a lower average RMSD than those created by alternative methods. Remarkably, RNAthreader takes less than two hours to complete alignments with ∼5000 RNAs, which is 3–40 times faster than other methods. These compelling results suggest that RNAthreader is a promising algorithm for RNA template detection.

**Availability and implementation:**

https://yanglab.qd.sdu.edu.cn/RNAthreader

## 1 Introduction

The 14th community-wide experiment on the critical assessment of techniques for protein structure prediction (CASP14) (Kryshtafovych *et al.* 2021) has witnessed great progress in protein structure prediction. With powerful deep learning techniques (Baek *et al.* 2021, Jumper *et al.* 2021), the structure prediction problem for single-domain proteins has been nearly solved (Peng *et al.* 2022). However, structure prediction for RNAs with deep learning remains challenging, due to the complexity of RNA molecules and the lack of enough experimentally solved structures (Wang *et al.* 2023). The RNA structure prediction methods can be broadly grouped into two categories: *ab initio* modeling and template-based modeling (TBM).

There are two major types of *ab initio* modeling methods. The first one is based on molecular dynamics simulation, such as Vfold3D (Zhao *et al.* 2017) and iFoldRNA (Krokhotin *et al.* 2015). The second one is based on fragment assembly, such as FARFAR (Das and Baker 2007), FARFAR2 (Watkins *et al.* 2020), RNAComposer (Popenda *et al.* 2012, Antczak *et al.* 2016), 3dRNA (Zhao *et al.* 2012, Zhang *et al.* 2022), and MC-Fold/MC-Sym (Parisien and Major 2008). These methods can be further grouped into full-atom simulation or coarse-grained simulation. The former performs a simulation of all atoms in a single round, which works well on short RNAs; while its great demand for computing resources prevents the application in structure prediction for large RNAs. The latter addresses this issue by merging certain atoms into so-called coarse-grained particles; while the ability to add atom-level details needs to be further strengthened.

TBM methods build the structure model for a query RNA using homologous templates in PDB (Berman *et al.* 2000). Accurate query-template alignment plays a key role in TBM methods. The alignment is usually conducted based on the secondary structure. However, RNA secondary structure is formed by base pairs (2D map), making the RNA alignment much more difficult than protein (1D sequence).

Sankoff proposed an algorithm to compute the alignment and the common secondary structure of two RNA sequences simultaneously, based on dynamic programming for free energy minimization (Sankoff 1985). This algorithm requires *O*(*n^6^*) time and *O*(*n^4^*) memory (*n* is the length of RNAs to be aligned), which grow very fast when the sequence length increases. This issue limits its real-world applications.

To address the above issue, several algorithms were proposed, which are referred to as Sankoff-style algorithms. There are three main types of Sankoff-style algorithms (Havgaard and Gorodkin 2014). The first is based on energy minimization, which is most similar to the original Sankoff algorithm, with representative methods such as Foldalign (Havgaard *et al.* 2007) and Dynalign (Mathews and Turner 2002). Methods of the second type are based on the alignment of base-pairing probability matrix, such as PMcomp (Hofacker *et al.* 2004) and LocARNA (Will *et al.* 2007). The third type of method uses statistical models based on stochastic context-free grammars (SCFG), such as Consan (Dowell and Eddy 2006) and RSEARCH (Klein and Eddy 2003). Some of these methods use different heuristic strategies to reduce time and memory consumption. For instance, Foldalign filters subalignments by setting length-related cutoffs, and LocARNA simplifies the base paring probability matrix by ignoring elements below a certain threshold.

There are some non-Sankoff-style algorithms for RNA alignment. For example, RNAmountAlign (Bayegan and Clote 2020) aligns two RNAs with Needleman-Wunsch dynamic programming (NWDP) (Needleman and Wunsch 1970) based on the mountain height (Hogeweg and Hesper 1984) of their secondary structures, and LARA (Bauer *et al.* 2007) is a graph-based method which solves the alignment problem by integer linear programming.

In this work, we developed a new RNA threading algorithm named RNAthreader. It is built on simplified secondary structure diagrams and sequence profiles from multiple sequence alignments. Benchmark tests show the advantage of RNAthreader over other methods in terms of both accuracy and speed.

## 2 Materials and methods

### 2.1 Template library

We retrieve experimentally determined 3D RNA structures from the RNAsolo database (Adamczyk *et al.* 2022) and use them to construct a template library comprising two distinct components: single-chain and multi-chain RNAs. The single-chain component encompasses all available single-chain RNA structures. Since base pairing between different chains is common, it is necessary to consider connecting different chains that form base pairs. To this end, we utilize RNAView (Yang *et al.* 2003) for secondary structure annotation, with the assembly structure as input. Specifically, for two chains denoted as A and B, if the number of base pairs between them exceeds 20% of the length of A or B, the combinations A_B and B_A will be added as new templates. In cases where a chain forms base pairs with multiple chains, we will include the additional chain that shares the largest number of base pairs with it. Consequently, each template in our library contains a maximum of three chains. Following this procedure, we successfully curate a template library comprising 18,195 RNAs. The library files are available for download at: https://yanglab.qd.sdu.edu.cn/RNAthreader.

### 2.2 Training and test datasets

Three datasets were used for training and testing. The training set, TR221, and the first test set, TE80, are obtained from our previous work RNAContact (Sun *et al.* 2020). TR221 consists of 221 RNAs, and TE80 comprises 80 RNAs, with sequence lengths spanning 32 to 1,000 nucleotides. RNAs from these two datasets share less than 30% sequence identity. Based on the presence or absence of pseudoknots, we further divided the TE80 dataset into two subsets: Knotted and Nested. Here a pseudoknot is a bipartite helical structure formed by base pairing of the apical loop in the stem-loop structure with an outside sequence (Peselis and Serganov 2014). The second test set (denoted by PUZ30) includes 30 RNAs from RNA-Puzzles (Magnus *et al.* 2019). Note that Puzzle 10, Puzzle 26, Puzzle 27, and Puzzle 28 are T-box riboswitch—tRNA complexes. For these targets, we only select T-box riboswitches for structure prediction. Besides, Puzzle 14 is an *L-glutamine riboswitch* with Bound and Free forms, which are treated as two different targets. More details about the PUZ30 dataset are given in Supplementary Table S1.

### 2.3 Algorithm inputs

#### 2.3.1 Sequence profile

The multiple sequence alignment (MSA) for each query RNA was generated with RNAcmap (Zhang *et al.* 2021). The RNAcmap method begins with an initial homolog search using BLAST-N (Altschul *et al.* 1997) against the NCBI nucleotide database. The returned homologs and the secondary structure predicted by SPOT-RNA (Singh *et al.* 2019) are then utilized to build a covariance model. In the end, an MSA is generated by the second round of homolog search using INFERNAL (Nawrocki and Eddy 2013). Given that the MSA may contain redundant sequences, we use HHfilter (Steinegger *et al.* 2019) to remove sequences sharing >80% sequence identity or <75% coverage with the query sequence. If the number of remaining homologs in the MSA is less than 5, we will not use sequence profiles in the following steps. Otherwise, we compute the sequence profile in a manner similar to that employed in PSI-BLAST (Altschul *et al.* 1997):

(1) $$F(i,k)={\;\text{log}\;}_{2}\frac{d(q_{i})f(i,k)+2p(q_{i})\sum_{m=1}^{4}2^{0.4R_{s}(q_{i},m)}f(i,m)}{p(q_{i})(d(q_{i})+2)},k=1,2,3,4$$

where *q_i_* is the nucleotide type for the *i*th position of the query RNA, *d*(*q_i_*) is the number of nucleotide types different from *q_i_* in the *i*th column of the MSA, *f*(*i*, *k*) is the observed frequency of the *k*th nucleotide in the *i*th column of the MSA, *p*(*q_i_*) is the background probability of *q_i_*, estimated from the NCBI nucleotide database (*p*(A) = 0.27, *p*(U) = 0.27, *p*(G) = 0.23, *p*(C) = 0.23) (Sun *et al.* 2018), and *R_s_* refers to the RIBOSUM single nucleotide matrix (Klein and Eddy 2003). Note that this profile automatically takes a balance between the MSA and the RIBOSUM matrix. The sequence profile largely depends on the MSA when it contains a large number of homologous sequences; while it resembles the RIBOSUM matrix when the MSA is shallow.

For RNAs in the template library, we also tried using BLAST-N for homologous sequence detection. However, many templates could not find any sequence homologs. To ensure the consistency of alignment scores, we did not use the sequence profile for templates. Thus, the query-template alignment will be performed in the profile-sequence or sequence-sequence manner in the following steps.

#### 2.3.2 Secondary structure

For the query RNA, we use the deep learning algorithm SPOT-RNA to predict its secondary structure. For RNAs in the template library, their secondary structures are extracted from native structures using RNAView. RNA secondary structure is usually represented as a planar graph (see Fig. 2A for an example).

### 2.4 Alignment algorithm

#### 2.4.1 Workflow of the alignment algorithm

**Figure 1.**
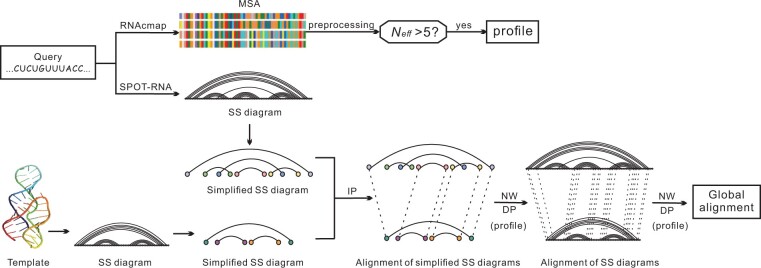
Flowchart of RNAthreader. Here “SS” stands for secondary structure, “IP” is integer programming, and “NWDP” denotes Needleman-Wunsch dynamic programming.

The flowchart of RNAthreader is presented in Fig. 1. It consists of two main steps. First, it extracts all the nucleotides forming base pairs and performs their alignments. Second, it generates the final alignment based on the sequence profile and the secondary structure alignment result.

#### 2.4.2 Alignment of simplified secondary diagrams

**Figure 2.**
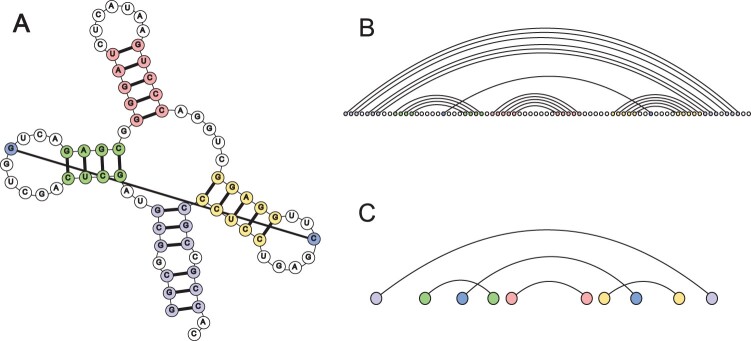
Secondary structure diagrams for an example RNA. (A) Planar graph with base pairs indicated by colored and connected vertices. (B) Linear arc diagram. (C) Simplified linear arc diagram.

The secondary structure of each RNA is converted into a linear arc diagram (Fig. 2B) for alignment. In the linear arc diagram, the vertices denote nucleotides and those connected by edges represent base pairs. With the graphical representation, the problem of finding the best alignment can be transformed into an integer programming problem with specified constraints (Bauer *et al.* 2007). However, the amount of constraints and variables in the integer programming problem grows rapidly with the increase of sequence lengths.

To address this issue, we simplify the linear arc diagram of RNA secondary structure as follows. First, only nucleotides forming base pairs are kept. Yet for RNAs with a large proportion of base pairs, this simplification only has a limited effect. Thus, we further define the concept of *substructures*. A substructure is a fragment consisting of consecutive base pairs, which allows the existence of bulge and interior loops (see Supplementary Fig. S1 for examples). As illustrated in Fig. 2A, the five colored fragments correspond to five different substructures. Second, we merge base pairs in the same substructure and get the final simplified diagram as shown in Fig. 2C. With the above procedures, the original diagram is significantly simplified and the relative positions of substructures are retained.

Initial alignment is then conducted on the simplified diagram. The alignment between two simplified diagrams is based on integer programming to maximize the following objective function.

(2) $$f=\sum_{\begin{array}{c} S_{i}^{q}\in V_{q}\\ S_{j}^{t}\in V_{t} \end{array}}\omega_{ij}\delta (S_{i,1}^{q},S_{j,1}^{t})\delta (S_{i,2}^{q},S_{j,2}^{t})$$

where *V_q_*/*V_t_* is the set of the substructures in the simplified diagram of the query/template; the substructure *Sq i* is denoted as a two-element tuple (*Sq i, 1*, *Sq i, 2*), in which *Sq i, 1* and *Sq i, 2* are the two paired halves; δ(*x*, *y*) equals to 1 if *x* and *y* are aligned, and 0 otherwise; *ω_ij_* is the matching score between substructures *Sq i* and *St j*, which is determined based on the NWDP (introduced in the next section). The objective function is subjective to two constraints to generate meaningful alignment: (1) there is no cross-alignment. In other words, once regions *x* and *y* are aligned, alignment cannot occur between the regions before *x* and the regions after *y* (or vice versa); (2) each vertex in a graph can be aligned to at most one vertex in another graph.

After solving the above integer programming problem, the alignment between two simplified secondary diagrams is obtained. It is used in the next step to generate nucleotide-level alignment with NWDP.

#### 2.4.3 Alignment of substructures with NWDP

The matching score and alignment between two substructures are obtained based on NWDP. Given that substructures only contain nucleotides forming base pairs, we design a *partial* alignment strategy, which only aligns half of the substructures. The alignment of the other half is completed automatically based on their base-pairing relationships. The scoring function for the partial alignment in NWDP is calculated as follows.

(3) $$\begin{array}{c} s(m_{1},n_{1})=\\ \{\begin{array}{c} R_{P}(q_{m_{1}}q_{m_{2}},t_{n_{1}}t_{n_{2}})+s_{E}((m_{1},m_{2}),(n_{1},n_{2})),\;\;\text{if}\;N_{\mathrm{eff}}<5\\ \frac{1}{2}[F_{q}(m_{1},t_{n_{1}})+F_{q}(m_{2},t_{n_{2}})]+s_{E}((m_{1},m_{2}),(n_{1},n_{2})),\;\;\text{if}\;N_{\mathrm{eff}}\geq 5 \end{array} \end{array}$$

(4) $$\begin{array}{c} s_{E}((m_{1},m_{2}),(n_{1},n_{2}))\\ =\{\begin{array}{c} 0,\;\text{if}\;E_{q}(m_{1},m_{2})\neq E_{t}(n_{1},n_{2})\\ 1,\;\text{if}\;E_{q}(m_{1},m_{2})=E_{t}(n_{1},n_{2})=(\text{stem},\;\text{stem})\\ 3,\;\;\text{otherwise} \end{array} \end{array}$$

where (*m_1_*, *m_2_*)/(*n_1_*, *n_2_*) is a base pair in a substructure of the query/template; *q_i_* is the type of the *i*th nucleotide in the query, *t_k_* is the type of the *k*th nucleotide in the template; *R_P_* refers to the RIBOSUM base-pairing matrix; *F_q_* is defined by Equation (1); *N_eff_* is the number of effective sequences in MSA, which equals to the number of remaining sequences in MSA after filtering; *s_E_* reflects the similarity of the base pairs in the local environment (Equation (4)). To define the local environment for a base pair (*i*, *j*), we first cut the whole diagram into two subgraphs at the edge (*i*, *j*). The environment for the base pair (*i*, *j*) in query is then defined as the smallest closed loop which starts at *i* and ends at *j* in each subgraph*.* Finally, the environment’s secondary structure type (stem, hairpin, bulge, interior loop, multi-loop, or pseudoknot, see Supplementary Fig. S1) makes the two-element tuple *E_q_*(*i*, *j*) in Equation (4).

If the sequence profile of the query RNA is not utilized, the gap open penalty is 3 and the gap extension penalty is 1. Otherwise, gap penalties will be adjusted according to the ratio of gaps in each column of the MSA, i.e. the higher the ratio of gaps is, the lower the gap penalty will be.

#### 2.4.4 Final alignment

From Section 2.4.2, we obtain the alignment between two simplified secondary diagrams. The nucleotide-level alignment between aligned substructures is the NWDP result described in Section 2.4.3. The final alignment between the query and each template is performed by NWDP with the following scoring function.

(5) $$s(i,j)=\{\begin{array}{c} R_{S}(q_{i},t_{j})+5\times I((i,j)\in S_{\mathrm{aln}}),\;\;\text{if}\;N_{\mathrm{eff}}<5\\ F_{q}(i,t_{j})+5\times I((i,j)\in S_{\mathrm{aln}}),\;\;\text{if}\;N_{\mathrm{eff}}\geq 5 \end{array}$$

where the definitions of *q_i_*, *t_j_*, *R_s_*, *N_eff_*, and *F_q_* are the same as those in Equation (1) and Equation (3); *I*(.) is an indicator function, whose value is 1 when the corresponding event happens, and 0 otherwise; *S_aln_* is the set of aligned nucleotides (*i*, *j*) in the previous step. The setting of gap penalties is the same as that described in Section 2.4.3 except that penalty is skipped for terminal gaps.

### 2.5 Performance evaluation

Alignment quality and model quality are used for evaluating the performance of different methods. Here the alignment quality is quantified by two metrics: alignment coverage and the RMSD of the aligned region. Alignment coverage is calculated by dividing the aligned length by the length of the query RNA. As for RMSD of the aligned region, we first extract coordinates of all heavy atoms on the backbone of aligned nucleotides from the template RNA, then compute RMSD with the corresponding region in the native structure. The other measure model quality is quantified by the RMSD between the native structure and the full-atom model built by FARFAR2 (Watkins *et al.* 2020) or ModeRNA (Rother *et al.* 2011) according to query-template alignment. The selection of the method for constructing the full-length model is determined by the alignment coverage. FARFAR2 is used when the alignment coverage varies significantly; otherwise, ModeRNA is used.

## 3 Results and discussions

### 3.1 Algorithm implementation and parameter optimization

The RNAthreader algorithm is implemented in C++. We use the high-performance mathematical programming engine IBM ILOG CPLEX 12.9 to solve the quadratic integer programming problem presented in Equation (2). With simplified linear arc diagrams of RNA secondary structures (Fig. 2C), the number of variables to be solved and corresponding constraints decrease substantially.

All the parameters were empirically selected to yield the best performance on the TR221 dataset. During the training process, templates sharing > 40% sequence identity with query RNAs were excluded. After the exclusion of close homologous templates, the average alignment coverage is relatively low. We tuned the parameters based on the alignment quality defined in Section 2.5.

### 3.2 Performance on the TE80 dataset

**Figure 3.**
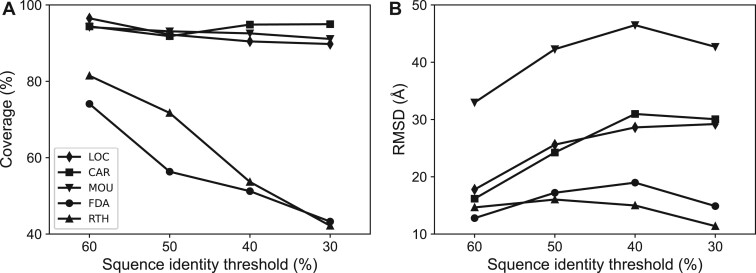
Alignment quality for the top-scoring alignment across different thresholds on the TE80 dataset. “LOC,” “CAR,” “MOU,” “FDA,” and “RTH” represent the methods LocARNA, CARNA, RNAmountAlign, Foldalign, and RNAthreader, respectively.

In the absence of dedicated RNA threading methods, we compare RNAthreader with four representative RNA alignment algorithms: LocARNA (version 1.9.2.1) (Will *et al.* 2007), CARNA (version 1.3.3) (Sorescu *et al.* 2012), Foldalign (version 2.5.3) (Sundfeld *et al.* 2015), and RNAmountAlign (Bayegan and Clote 2020). We first conduct experiments on the TE80 dataset with four distinct thresholds: 60%, 50%, 40%, and 30%, which correspond to four progressively increasing levels of threading difficulty. In each comparison, templates with sequence identity to the query exceeding a certain threshold are excluded. The average number of non-redundant templates (<99% pairwise sequence identity) remaining in the library for the four thresholds are as follows: 3991, 3713, 3344, and 2817, respectively. Figure 3 depicts the alignment quality, assessed in terms of coverage and RMSD, for the top-scoring alignment across different thresholds.

The compared methods can be categorized into two groups based on the average coverage (as shown in Fig. 3A): LocARNA, CARNA, and RNAmountAlign consistently maintain high coverages across different thresholds, while Foldalign and RNAthreader exhibit increasing average coverages as more templates with higher sequence identity become available. Likewise, there is a notable variation in RMSDs between these two groups of methods (Fig. 3B). In the first group, the RMSDs of these methods have a tendency to increase as the threshold decreases, indicating that they could not effectively identify structure templates with low sequence identity. Moreover, RNAmountAlign yields alignments with significantly higher RMSDs when compared to LocARNA and CARNA. This observation suggests that it might not be appropriate to directly apply the Needleman-Wunsch dynamic programming to align RNAs. As for methods in the second group, namely Foldalign and RNAthreader, they exhibit relatively low average RMSDs that remain consistent regardless of the threading difficulty. The above facts confirm the reliability of these two methods in template detection.

For proteins, a remote homolog is typically defined as a homologous template with less than 30% sequence identity to the query (Khor *et al.* 2015). However, due to the limited number of nucleotide types compared to amino acids, RNA sequences often exhibit higher similarity. Therefore, for RNAs, we use a threshold of 40% for remote homologs and conduct a more detailed analysis at this specific threshold.

For each query, the alignment coverage for different methods varies, making the direct comparison in terms of RMSD less meaningful. To address this issue, we say method A generates more accurate alignment than method B on a target if the following two conditions are satisfied: (1) the RMSD on the aligned region of method A is lower than that of method B; (2) the alignment coverage of method A is higher than that of method B. The comparisons of the methods in the two above-mentioned groups are available in Supplementary Figs S2 and S3, respectively. Our method outperforms Foldalign on 24 targets in the set TE80; while Foldalign outperforms our method on 9 targets.

Given the diverse coverage of alignments from different methods, we integrated template information into FARFAR2, a fragment assembly-based approach for RNA structure prediction, to enhance the assessment of the generated alignments. Specifically, the structure of the aligned region from the top-scoring template, coupled with the query sequence and the secondary structure predicted by RNAfold (Lorenz *et al.* 2011), served as inputs for FARFAR2. If FARFAR2 fails to build models for a template, then the next-ranked template will be utilized, until a model is built successfully. Table 1 displays the RMSD values and the count of optimal models (those with the lowest RMSD value) for the generated structural models using templates with <40% sequence identity.

**Table 1.** RMSD and optimal model count (#) for FARFAR2 models using top-scoring templates with <40% sequence identity.

Method	All (80)		Knotted (33)		Nested (47)	
	RMSD(Å)	#	RMSD(Å)	#	RMSD(Å)	#
LOC	29.1	11	32	3	27	8
CAR	29.3	19	32.9	8	26.8	11
MOU	40.9	4	48.4	3	35.7	1
FDA	26.2	16	30.4	7	23.3	9
RTH	21.4	30	23.1	12	20.2	18

“LOC,” “CAR,” “MOU,” “FDA,” and “RTH” represent the methods LocARNA, CARNA, RNAmountAlign, Foldalign, and RNAthreader, respectively.

Based on the data presented in Table 1, models generated with templates selected by RNAthreader exhibit the lowest average RMSD on the TE80 dataset, which is 18.3% lower than that of the next-best method, Foldalign. Additionally, the RNAthreader model outperforms all other compared methods by achieving the lowest RMSD among 30 targets, indicating its superior performance. We also compared the results with the original FARFAR2 model, which was generated solely using sequence and predicted secondary structure as inputs. The average RMSD (20.9 Å) of the original FARFAR2 model is slightly lower than that of RNAthreader. Despite this, when considering the number of best models, the original FARFAR2 method achieves the best performance on 18 targets, while RNAthreader performs best on 21 targets. The remaining four methods, namely LocARNA, CARNA, RNAmountAlign, and Foldalign, yield results of 8, 16, 4, and 13, respectively.

We then divide the test set into two subsets, Knotted and Nested, to compare all methods further. The average RMSD for RNAthreader models is 24% lower than that of the second-best method Foldalign on the Knotted dataset, demonstrating the advantage of our method on RNAs with pseudoknots. Besides, within the Knotted subset, we further compute the average coverage of alignments on pseudoknotted base pairs. The results for LocARNA, CARNA, RNAmountAlign, Foldalign, and RNAthreader are 85.6%, 95.8%, 92.6%, 12.8%, and 18%, respectively.

Finally, we assess the influence of skipping terminal gap penalties on RNAthreader’s performance. It turns out that omitting penalties for terminal gaps has a negligible impact on alignment coverage; however, it results in a noticeable improvement in the corresponding RMSD values. For instance, at the 40% threshold, the average alignment coverage remains nearly consistent, whether or not gap penalties are omitted (53.7% verus 53.5%). Nevertheless, the RMSD decreases from 17.6 Å to 15 Å when penalties for terminal gaps are skipped.

### 3.3 Performance on the RNA-Puzzles targets

We further test our method on the RNA-Puzzles targets. To simulate the situation of the RNA-Puzzles experiments, for targets in the PUZ30 dataset, we excluded all templates released after 1 January 2010. Table 2 shows the alignment quality (RMSD and Coverage) on the aligned region and the RMSD of the full-length models. Given high alignment coverage, the full-length model is built by the homology modeling method, ModeRNA, with default parameters based on the top-scoring template. Similarly, if ModeRNA fails to build models for a target, the next template will be utilized until a model is successfully built.

**Table 2.** Performance of different methods on the RNA-Puzzles targets.

Method	Aligned region		Full-length model
	RMSD (Å)	Coverage (%)	RMSD (Å)
LOC	20.9	96	20.3
CAR	19.4	94.4	21
MOU	23.9	94.3	25.4
FDA	16.4	75.1	23.6
RTH	17.6	81.2	20.1

From Table 2, we find that besides LocARNA, CARNA, and RNAmountAlign maintaining over 90% coverage, Foldalign and RNAthreader also achieve relatively high alignment coverage. RMSDs for other methods increase for the full-length models, except for LocARNA, which has the highest coverage. Additionally, Foldalign shows the largest increase in RMSD with a rise of 44.1%.

**Figure 4.**
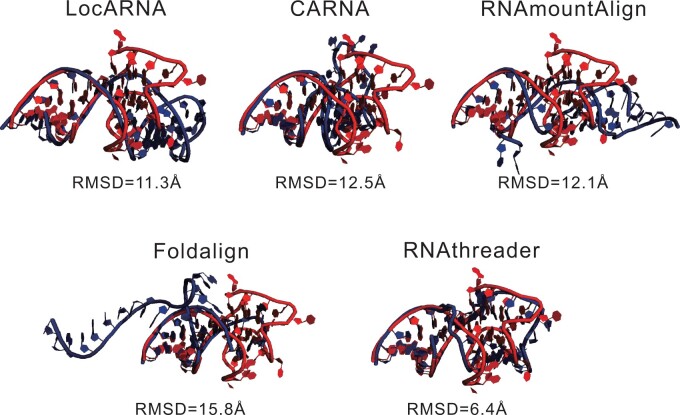
Superimposition of the predicted models (in blue) against the native structure (in red) for an example RNA-Puzzles target Puzzle 23 (PDB ID: 6E8U).

Even if the average coverage of RNAthreader is not the highest, the quality of the full-length models generated by ModeRNA with templates selected by RNAthreader is the best among all the methods. In Fig. 4, we present an example target from RNA-Puzzles (Puzzle 23, PDB ID: 6E8U) that RNAthreader outperforms other methods. This particular example involves a *mango-III aptamer* (Trachman *et al.* 2019). For this target, the RMSDs of the full-length models built using alignments generated by LocARNA, CARNA, RNAmountAlign, Foldalign, and RNAthreader are 11.27, 12.53, 12.07, 15.8, and 6.38 Ås, respectively. The model of RNAthreader is significantly more accurate than that of other methods, whose template 2OE5_B_A is the combination of two chains of 2OE5, with a coverage of 81.1%. Indeed, according to the assessment results released on the RNA-Puzzles website, the best model in the competition is from the Das Lab, with an RMSD of 8.129 Å, which is slightly worse than our model.

### 3.4 Analysis of multiple sequence alignment

Unlike conventional RNA alignment methods, RNAthreader utilizes sequence profiles calculated from MSA. As described above, we filter each MSA based on coverage and sequence identity. If more than 5 homologous sequences remain in the preprocessed MSA, then a sequence profile calculated by Equation (1) will be utilized. After filtering, the sequence profile is used for 60 RNAs in the TE80 dataset when searching for templates. To study the impact of MSA utilization, we compare the performance of three different ways of using MSAs, i.e. not using MSAs (denoted by “noMSA”), using original MSAs generated by RNAcmap (denoted by “originMSA”), using filtered MSAs (denoted by “filterMSA”) on these 60 RNAs. The results after excluding templates with >40% sequence identity are summarized in Supplementary Fig. S4.

Supplementary Fig. S4A and B show the comparison in terms of the alignment quality. Compared with not using MSAs, the coverage of the alignment increases by 2.8% after the inclusion of origin MSAs. The average RMSD increases as well, illustrating that the contribution from the original MSA is less significant. However, when filtered MSA is utilized for the sequence profile calculation, the alignment quality improves, with an increase of 7.6% in average coverage and a decrease of 2.6% in RMSD. This demonstrates the importance of filtering MSA. Supplementary Fig. S4C shows the comparison in terms of the number of better alignments. With filtered MSA, RNAthreader generates more accurate alignments (higher coverage and lower RMSD) on more targets than not using MSA and using the original MSA. In particular, there are 15 RNAs on which the “filterMSA” has higher alignment quality than “noMSA,” while the latter does not have any alignments superior to the former.

### 3.5 Analysis of the predicted secondary structure

The accuracy of secondary structure prediction may have a direct impact on the performance of RNAthreader, as the predicted secondary structure is an essential component of the alignment progress.

Note that among the compared methods, only two methods CARNA and RNAthreader, are capable of handling pseudoknots. Hence, we first aim to verify whether RNAthreader's performance is primarily influenced by improved secondary structure prediction. To this end, we utilize RNAfold (Lorenz *et al.* 2011), a minimum free energy (MFE) based approach, to predict secondary structure and then incorporate its output as input into RNAthreader. It turns out that after excluding homologous templates with >40% sequence identity, RNAthreader produce alignments with an average RMSD of 16.2 Å and a coverage of 50.5% on the TE80 dataset. Additionally, the accuracy of predicted secondary structures, as assessed by the average interaction network fidelity (INF) (Parisien *et al.* 2009), is 0.71 for SPOT-RNA and 0.62 for RNAfold, respectively. The results show that as the quality of RNA secondary structure prediction decreases, the performance of RNAthreader indeed experiences a decline. However, it's noteworthy that RNAthreader consistently generates a larger quantity of superior alignments (with lower RMSD and higher coverage) compared to Foldalign, another method capable of detecting remote homologous templates. In fact, RNAthreader produces superior alignments to Foldalign on 16 RNAs, while Foldalign outperforms RNAthreader on 7 RNAs.

Subsequently, we further explore how the quality of secondary structure affects the three-dimensional structural models. For the PUZ30 dataset, the Pearson’s correlation coefficient (PCC) between INFs of secondary structures predicted by SPOT-RNA and RMSDs of structure models is −0.622. Supplementary Fig. S5 shows the scatter plot between the INF and the RMSD, suggesting that the more accurate secondary structure predictions do lead to more accurate 3D structure models from threading.

### 3.6 Running time analysis

We compare the speed of different methods by collecting their running time of searching against the same template library. Given that some of the methods being compared are extremely time-consuming, we exclude redundant sequences from the original library at 99% sequence identity. Then the following experiment was performed on this non-redundant library with 4860 RNAs.

Supplementary Fig. S6 and Supplementary Table S2 show the running time of different methods on targets from the TE80 dataset. For better visualization, the longest RNA in this dataset (PDB ID: 5WZN_P) is excluded in Supplementary Fig. S6. We can see from the figure that LocARNA has the highest growth rates in running time as the sequence length increases, followed by CARNA and Foldalign. In contrast, the speed of RNAmountAlign and our method RNAthreader do not have obvious changes. The average running time per RNA for LocARNA, CARNA, RNAmountAlign, Foldalign, and RNAthreader on the TE80 dataset is 42.3, 19.9, 8.6, 4.5, and 1.3 h, respectively. The high speed of our method compared with others is mostly due to the use of the simplified linear arc diagram.

## 4 Conclusion

We have developed a novel RNA threading algorithm named RNAthreader using RNA secondary structure and sequence profile. It speeds up the alignment significantly with a reduced representation of RNA secondary structure and improves the alignment accuracy with the sequence profile. Benchmark tests show that RNAthreader outperforms other methods in terms of alignment accuracy and speed. Nevertheless, we admit that the models built from the RNAthreader templates are not perfect. This may be because of the limited size of the existing RNA template library. We anticipate RNAthreader could be improved further with the release of more experimental RNA structures in the future. Another promising way to improve is using the powerful deep learning algorithms.

## Supplementary Material

btae080_Supplementary_Data

## Data Availability

The data underlying this article are available at https://yanglab.qd.sdu.edu.cn/RNAthreader/.
