# Supplementary material for: RNA threading with secondary structure and sequence profile
Source: Bioinformatics. 2024 Feb 10;40(2):btae080. doi: 10.1093/bioinformatics/btae080 (PMC10893584; doi:10.1093/bioinformatics/btae080)
Supplement: btae080_Supplementary_Data [file btae080_supplementary_data.pdf]

Supplementary Materials

Table S1 Targets in the PUZ30 dataset.

	PDB ID	Type
Puzzle 1	3MEI	Regulatory motif from the thymidylate synthase mRNA
Puzzle 3	3OWZ	Glycine riboswitch
Puzzle 4	3V7E	SAM-I riboswitch aptamer
Puzzle 5	4P9R	Group I intron
Puzzle 6	4GXY	Adenosylcobalamin riboswitch
Puzzle 7	4R4V	Varkud satellite ribozyme
Puzzle 8	4L81	SAM riboswitch
Puzzle 9	5KPY	5-hydroxytryptophan aptamer
Puzzle 10	4LCK (Chain C, F)	T-box riboswitch
Puzzle 11	5LYS, 5LYU, 5LYV, 5LEM	7SK
Puzzle 12	4QLM	ydaO riboswitch
Puzzle 13	4XW7	ZMP Riboswitch
Puzzle 14 Bound form	5DDO	L-glutamine riboswitch
Puzzle 14 Free form	5DDO	L-glutamine riboswitch
Puzzle 15	5DI4	Hammerhead Ribozyme
Puzzle 17	5K7C	Pistol ribozyme
Puzzle 18	5TPY	Zika virus
Puzzle 19	5T5A	One Twister Sister robozyme
Puzzle 20	5Y85, 5Y87	Another Twister Sister ribozyme
Puzzle 21	5NWQ, 5NZ6	Guanidine III Riboswitch
Puzzle 22	6JQ5, 6JQ6	Hatchet Ribozyme
Puzzle 23	6E8U	Mango-III aptamer
Puzzle 24	6OL3	Adenovirus virus-associated RNA
Puzzle 25	6P2H	2'-dG-II riboswitch
Puzzle 26	6PMO (Chain B)	T-box riboswitch
Puzzle 27	6POM (Chain A)	T-box riboswitch
Puzzle 28	6UFM (Chain B)	T-box riboswitch
Puzzle 29	6TB7	NAD+ riboswitch
Puzzle 30	7BG9	Human telomerase RNA
Puzzle 33	7ELP, 7ELQ, 7ELR, 7ELS	Xanthine riboswitch

Table S2 Running time (hour) of different methods searching against the template library for targets in the TE80 dataset.

	Length	LocARNA	CARNA	RNAmountAlign	Foldalign	RNAthreader
6C63_A	36	13.06	6.13	4.33	0.42	0.96
1KOG_O	37	48.07	4.07	13.93	0.49	1.07
1F1T_A	38	47.73	3.51	14.46	0.5	1.14
3P22_A	40	14.79	2.87	14.01	0.5	1
5GAH_1	43	16.25	9.75	4.5	0.58	0.97
1P6V_B	45	51.12	6.45	14.93	1.5	1.06
2NUE_C	46	15.7	4.89	14.09	0.61	1.08
4O26_E	47	16.34	5.21	4.11	0.63	1.13
5DCV_D	47	16.39	10.24	4.31	0.62	1.07
4C7O_E	48	15.3	19.13	4	1.06	1.06
1U63_D	49	50.12	10.45	14.49	0.96	1.19
3NPQ_A	51	15.16	2.31	14.29	0.99	1
6AAY_B	52	21.8	7.95	4.08	1.04	0.76
4GCW_B	55	16.78	22.39	4	0.77	1.96
5OOL_B	56	18.34	4.64	4.54	0.82	0.68
4V2S_Q	58	15.94	17.35	4.35	0.82	1.25
4V8Q_BY	61	16.39	5.07	4.27	1.01	0.94
5T5A_A	61	19.34	2.59	4.61	1.38	0.68
2CZJ_H	62	25.84	11	14.69	1.13	1.18
3RW6_H	62	16.38	14.81	3.98	1.27	0.99
4YCP_B	62	19.84	13.38	4.49	1.56	1.3
1Y27_X	68	49.07	20.74	13.36	1.2	1.2
3SKI_A	68	18.88	17.9	4	1.48	1.2
3EPH_E	69	21.16	16.09	14.15	2.27	1.28
6ASO_I	69	29.52	8.8	4.09	1.23	0.76
1KXK_A	70	56.45	25.92	13.94	2.15	1.07
2DER_D	71	18.8	14.76	14.61	2.47	1.29
2DU3_D	71	17.71	16.68	14.6	1.84	1.38
2OIU_Q	71	18.41	23.02	14.55	2.28	1.31
4PR6_B	72	15.33	19.06	4.37	2.79	1.26
5WWT_C	72	23.4	19.89	3.86	1.86	1.68
1U0B_A	74	65.73	13.63	15	1.8	1.2
4PRF_B	74	15.19	14.57	4.36	3.32	1.26
4WQ1_3K	74	18.08	19.31	4.42	1.7	1.06
5D8H_A	74	18.96	11.9	4.5	1.91	0.98
6OKK_g	74	18.23	19.37	4.34	1.32	1.4
1F7U_B	75	59.5	26.13	14.42	2.94	1.73
1GAX_D	75	62.54	20.12	14	3.07	1.62
1N78_C	75	49.66	19.63	14.16	2.96	1.34
2CSX_C	75	63.96	19.05	14.68	2.5	1.67
4LCK_B	75	17.09	15.49	4.11	2.72	1.31
4V7M_AY	75	18.44	21.38	4.33	2.85	1.3

6FYY_1	75	17.66	19.87	4.3	1.66	1.52
1J2B_C	77	65.08	23.6	14	2.7	1.74
4V9Q_DV	77	19.79	21.93	4.62	2.53	1.49
5O2R_x	77	22.51	20.08	4.56	2.77	1.21
2BTE_B	78	72.3	15.26	12.93	3.08	1.19
3AMU_B	78	18.25	19.22	22.4	2.35	1.52
5VPP_QV	78	22.48	16.83	3.89	3.06	1.08
3U4M_B	80	21.4	24.92	4.13	3.12	1.32
6JQ5_B	81	16.06	10.3	4.25	2.25	1.21
3Q1Q_C	86	17.13	19	15.22	3.65	1.48
1WZ2_D	88	110.4	20.63	13.38	4.49	1.46
1M5K_B	92	69.52	9.5	14.15	3.05	1.07
6AZ3_5	97	44.08	16.79	3.89	3.63	1.41
3W3S_B	98	18.74	26.79	4.15	5.65	1.32
6CB3_B	99	17.91	22.79	4.35	3.64	1.14
2XXA_F	102	38.23	24.86	22.23	5.76	1.37
6QW6_5	104	19.66	20.68	4.41	3.68	0.91
3JB9_C	105	20.24	26.52	14.3	3.92	1
6CK5_A	108	21.58	21.58	4.42	4.3	1.13
6OL3_C	111	21.22	26.81	4.44	7.43	1.21
4WF9_Y	114	25.22	32.96	4.44	3.73	0.98
5T5H_D	114	39.03	29.01	4.65	4.99	0.92
6AZ3_8	118	29.84	32.5	3.89	5.51	0.73
6RM3_L70	118	20.85	33.99	4.27	3.26	0.91
4V8P_C3	120	26.91	34.64	4.27	6.15	0.97
5J7L_DB	120	36.6	31.17	4.6	5.37	0.77
2IL9_A	135	27.25	23.03	14.21	5.28	1.28
3G9C_Q	141	23.4	24.62	14.15	7.11	1.59
6G90_2	143	19.03	29.58	4.35	7.69	1.06
5T5H_E	146	67	26.81	4.42	5.23	1.67
1U9S_A	155	166.47	27.9	14.51	11.79	1.94
2R8S_R	159	35.02	22.88	14.65	9.34	1.26
3P49_A	169	87.69	36.78	14.07	9	1.53
6J6G_D	179	29.79	21.55	4.48	8.52	1.22
4R4V_A	186	54.21	38.67	4.71	13.69	2.27
5FLX_z	264	157.97	40.57	4.55	36.37	5.15
2A64_A	298	202.95	55.99	13.24	32.97	3.04
5ZWN_P	480	492.98	71.44	5.15	44.16	1.66

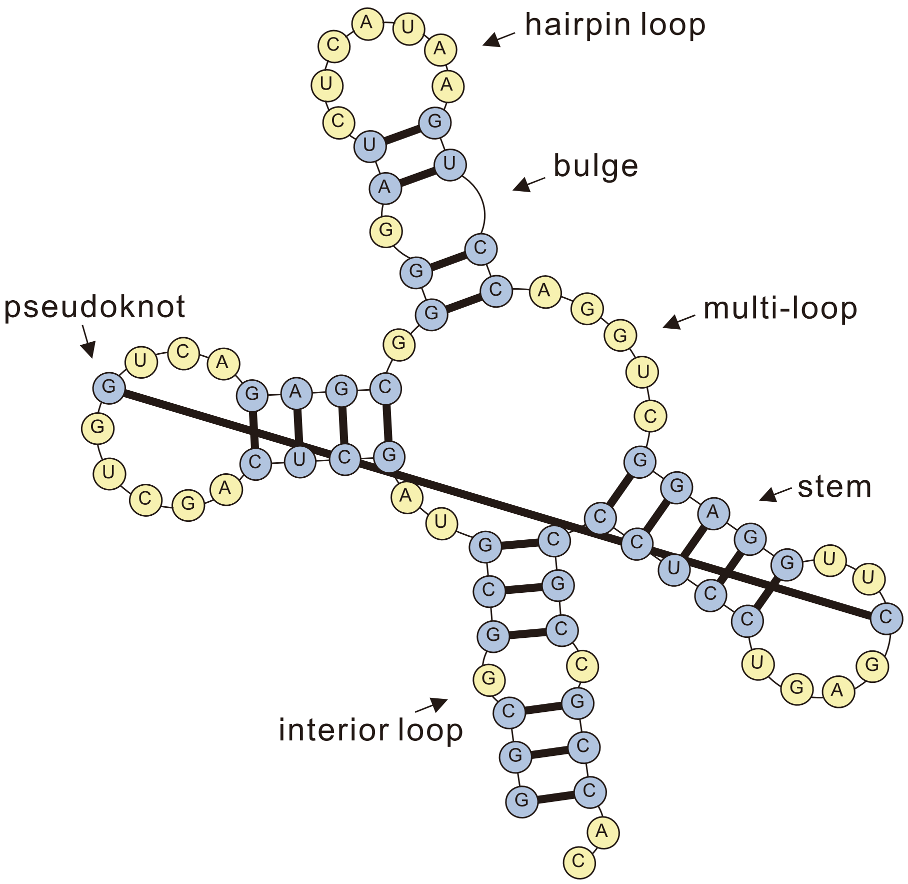

Figure S1 Secondary structure types of RNAs.

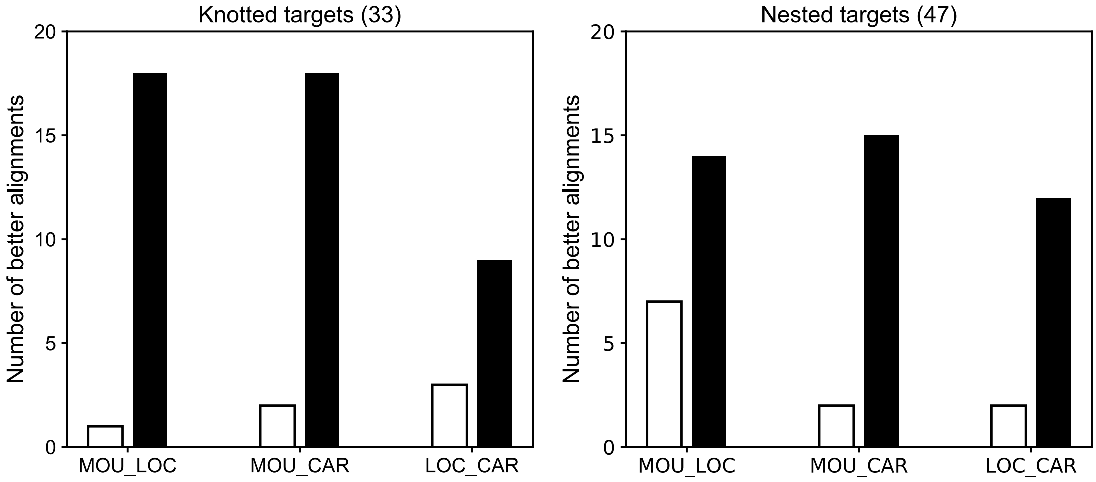

Figure S2 Comparison between LocARNA, CARNA, and RNAmountAlign in terms of the number of better alignments on two subsets of the TE80 dataset after excluding templates sharing >40% sequence identity with the query. 'LOC', 'CAR', and 'MOU' represent the methods LocARNA, CARNA, RNAmountAlign, respectively. Here 'A_B' refers to the comparison between method A and method B, and the height of a white/black bar is the number of targets on which method A/B generates better alignments than method B/A.

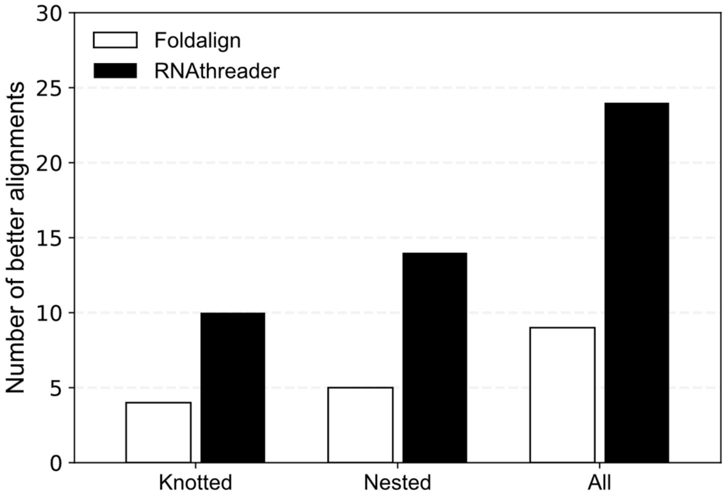

Figure S3 Comparison between Foldalign and RNAthreader after excluding templates sharing >40% sequence identity with the query. The height of each bar is the number of targets with more accurate alignment. The compared dataset is TE80 and its subsets.

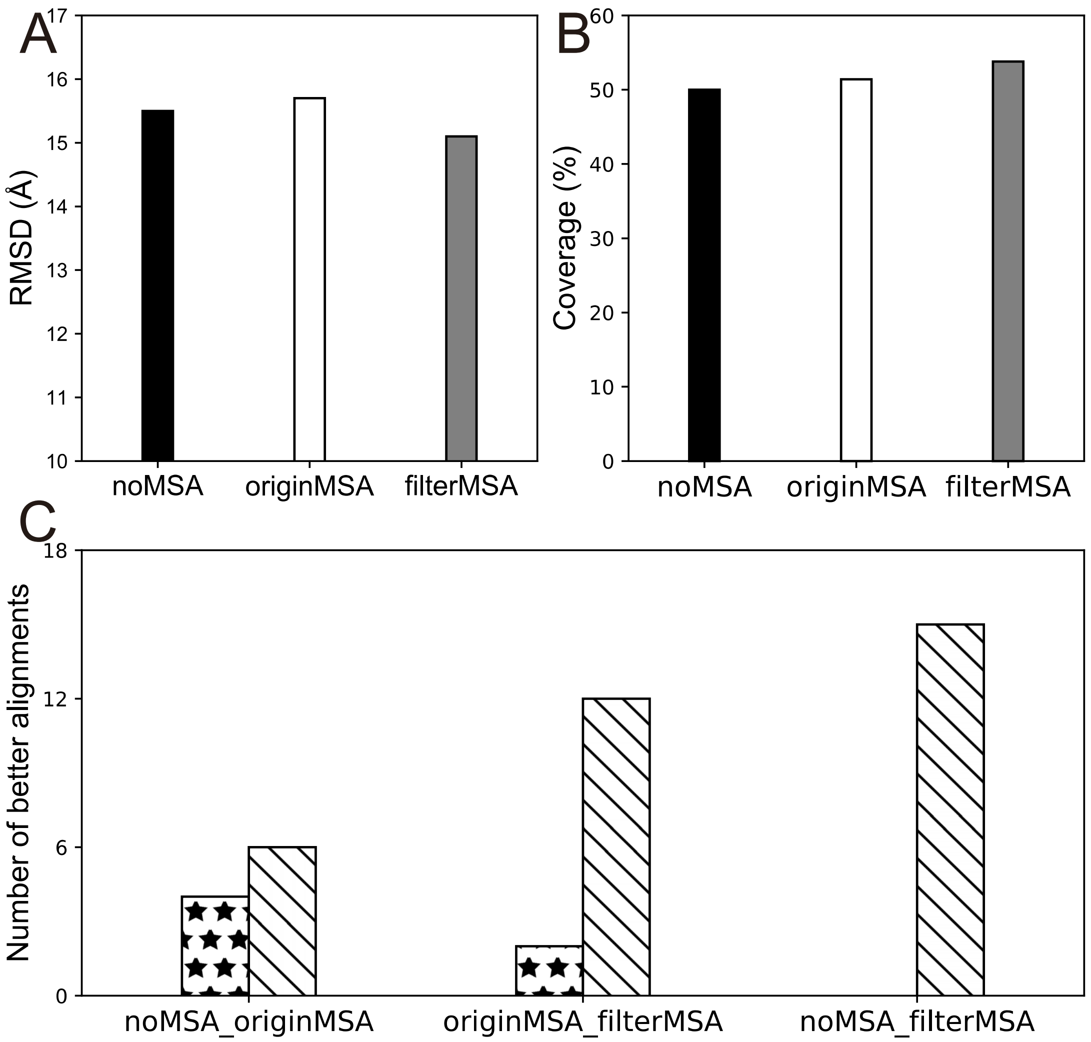

Figure S4 Comparison between different ways of using the MSAs on 60 targets from the TE80 dataset after excluding templates sharing >40% sequence identity with the query. (A) The average RMSD of the aligned region. (B) The average coverage of alignments. (C) The comparison in terms of the number of better alignments. Here 'A_B' refers to the comparison between method A and method B, and the height of a bar filled with stars/slashes is the number of targets on which method A/B generates better alignments than method B/A.

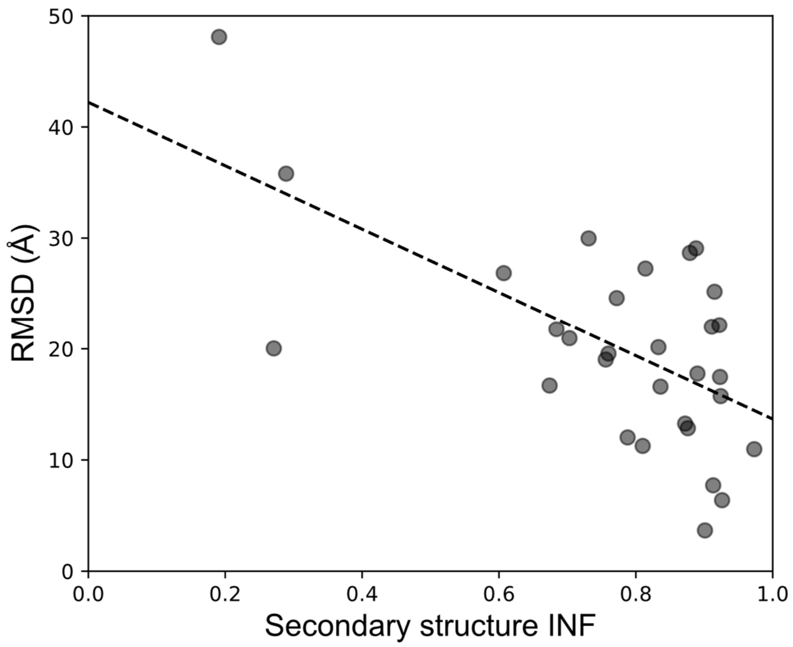

Figure S5 Scatter plot between the model accuracy and the interaction network fidelity (INF) of secondary structure. The data are based the on the RNA-Puzzles targets.

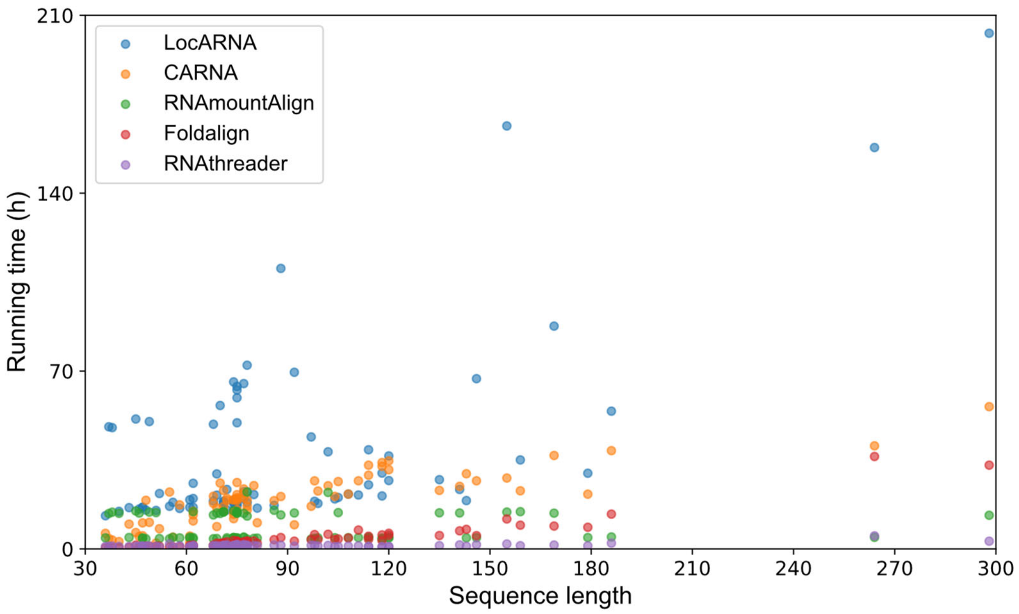

Figure S6 Running time (hour) of different methods searching against the template library for targets in the TE80 dataset.
